# Book Notices

**Published:** 1897-04

**Authors:** 


### Book Notices.

ESSENTIALS OF PHYSICAL DIAGNOSIS of the Thorax: by Arthur M. Corwin, A. M., M. D., Demonstrator of Physical Diagnosis in Rush Medical College, etc. Second Edition, revised and enlarged. Price, in cloth, $1.25. W. B. Saunders, Publisher, 925 Walnut Street, Philadelphia.

This little volume of nearly 200 pages, covers the main points in the physical examination of the thorax. It is arranged in a systematic form, and the student will find it a valuable aid in the study of physical diagnosis. S. E. H.

MANUAL OF GYNECOLOGY. By Henry T. Byford, M. D., Professor of Gynecology and Clinical Gynecology, in the College of Physicians and Surgeons of Chicago; Professor of Clinical Gynecology in the Woman's Medical School of Northwestern University; Professor of Gynecology in the Post-Graduate Medical School of Chicago. 488 pages, 234 illustrations, many of which are original. Price, in cloth, $2.50. P. Blakiston, Son & Co., Publishers, 1012 Walnut St., Philadelphia.

This manual will be very useful to the medical student during his college course and first years of practice; also to the older general practitioner, who realizes that he cannot attempt to manage the more complicated cases, or perform the more difficult operations. The book is written from the standpoint of a teacher and practitioner, and has the especial merit of being pointed and practical. The arrangement of the subject matter has also the advantage of maintaining the students' interest better than that found in most text-books. There are few repetitions, owing to the arrangement of the subject matter and the author's method of references. Much space is saved in this way, and corresponding time saved to the student.

S. E. H.

---

INEBRIETY; its source, prevention, and cure, by Charles Follen Palmer. Price, in cloth, with gilt top, 50 cents. Fleming H. Revell Co., Publishers, New York, Chicago and Toronto.

The author treats of inebriety as a disease, and gives the various causes, inheritance, perverted sensations, etc. In the matter of treatment he recommends no specific medication, but such agents as will build up the general health, together with proper surroundings, the building up of a healthy will power and moral character, and in some cases he recommends confinement in an inebriate asylum. The volume is a very interesting and instructive one, and is well worth a careful perusal. S. E. H.

---

THE YEAR-BOOK OF TREATMENT FOR 1897. A Critical Review for Practitioners of Medicine and Surgery. Crown octavo, 488 pages. Cloth, $1.50. Philadelphia and New York. Lea Brothers & Co., 1897.

This is a compact little volume, carefully prepared, and covers the more important advances made in medicine and surgery during the past twelve months.

The list of contributors number twenty-six, each one having a special subject or subjects. The whole field of late medical literature, including monographs, journals and text-books, has been searched, and that which is practical and useful has been culled, and condensed into the fewest possible words. This conciseness adds much to the real value of the volume.

S. E. H.

---

THE INTERNATIONAL MEDICAL ANNUAL AND PRACTITIONER'S INDEX. A work of Reference for Medical Practitioners, by various European and American contributors. 724 pages, and numerous full page plates and engravings. Fifteenth year. Price, in cloth, $2.75. E. B. Treat Publisher, 241, 243 West 23d St., New York City. 1897.

"Treat's Annual" has been so long before the profession, and has occupied so prominent and favorable a position that it is almost useless to attempt an introduction. The present edition is not behind its predecessors in any respect, and in one particular, viz: the larger number of American contributors, it will be more acceptable to the profession of this country than the editions of former years. The contributors to this year's "Annual" number forty-one, among them we note Prof. Augustus Caille, M. D., Prof. Henry D. Chapin, M. D., Prof. Wm. S. Gotthiel, M. D., Prof. Græme, M. Hammond, M. D., Irving S. Haynes, M. D., Frank W. Jackson, M. D., all of New York; Prof. Theophilus Parvin, M. D., Prof. George E. de Schweinitz, M. D., Clarence A. Veasey, M. D., of Philadelphia; John Ridlon, M. D., of Chicago. Of the more noted foreign contributors, we name Wm. Murrell, M. D., Joseph Priestley, M. D., Herbert W. Allingham, F. R. C. S., E. Hurry, Fenwick, F. R. C. S., J. Dundas Grant, M. D., Alex. Haig, M. D., of London; Robert Saundby, M. D., of Birmingham; A. W. Mayo Robson, F. R. C. S., of Leeds; Wm. Thornburn, F. R. C. S., of Manchester; Prof. E. Sonnenburg, M. D., of Berlin; Norman Walker, M. D., of Edinburg; David Hardie, M. D., of Brisbane; Prof. P. G. Unna, of Hamburg; Thos. Moore Maddin, M. D., of Dublin; G. Armauer Hansen, M. D., of Bergen, and a number of other noted names. With this list of famous physicians as contributors, it is hardly necessary to add that this volume represents the best of medical progress throughout the world during the past year. S. E. H.

---

An American Text-Book of Obstetrics for Practitioners and Students. By James C. Cameron, M. D., Edward P. Davis, M. D., Robert L. Dickinson, M. D., Charles Warrington Earle, M. D., James H. Etheridge, M. D., Henry J. Garrigues, M. D., Barton Cooke Hirst, M. D., Charles Jewett, M. D., Howard A. Kelley, M. D., Richard C. Norris, M. D., Chauncey D. Palmer, M. D., Theophilus Parvin, M. D., George A. Piersol, M. D., Edward Reynolds, M. D., Henry Schwarz, M. D. Richard C. Norris, M. D., editor, Robert L. Dickinson, M. D., Art editor. In one royal octavo volume of 1009 pages, with nearly 900 colored and halftone engravings. Price, cloth, $7.00; sheep, $8.00; half russia, $9.00. For sale by subscription only. W. B. Saunders, publisher, 925 Walnut Street, Philadelphia.

This book embodies the teachings of a number of prominent American obstetricians, possessing experience as teachers of obstetrics in several of the leading medical schools and hospitals of this country. As we would expect from such a list of authors, it reflects all the recent progress made in the theory and practice of obstetrics, and is an admirable text-book for students and a guide for practitioners. Especial prominence has been given to the practical part of obstetrical work, obstetric emergencies, the mechanics of normal and abnormal labor, and the various manipulations required in obstetric surgery are all described in great detail. More prominence is given to diseases of the fetus and the new-born infant than is usual in obstetrical works. In the illustrations all figures have been drawn to a uniform scale, usually one-third or one-sixth life size, and in sagittal sections the same half is always shown for ease of comparison. Full labeling is made directly on the drawing. The text throughout is liberally illustrated with full page plates, drawings and diagrams, which will materially aid the student in grasping the complex problems of operative obstetrics. The directions for treatment are particular and full, making of this work a very satisfactory guide to those who practice obstetrics.

S. E. H.

---

A Text-Book of Materia Medica, Therapeutics and Pharmacology. By George Frank Butler, Ph. G., M. D., Professor of Materia Medica and Clinical Medicine in the College of Physicians and Surgeons, Chicago; Professor of Materia Medica and Therapeutics, Northwestern University, Woman's Medical School; Attending Physician to Cook County Hospital, etc., etc., etc. In one volume of 858 pages. Price, cloth, $4.0C net. W. B. Saunders, Publisher, 925 Walnut St., Philadelphia.

This is a concise, practical text-book, especially serviceable to the medical student. The author has excluded many of the newer drugs whose therapeutic value is not yet fully established, and a number of the older ones that are practically never used or are employed only in isolated cases.

The arrangement is a very convenient one for the student beginning with a chapter on pharmacology and general therapeutics. This is followed by a chapter on pharmaceutical preparations, embracing solutions, liquid mixtures, extractive preparations, solid mixtures for internal use, and preparations for external use. The various official and many non-official preparations are discussed in this chapter, including medicated waters, solutions, spirits, syrups, elixirs, emulsions, infusions, decoctions, wines, tinctures, fluid and solid extracts, resins, powders, confections, pills, liniments, washes, ointments, plasters, bandages, dressings, etc.

The discussion of medicinal agents proper, he has divided into the following classes: Class I. Disease medicines, including restoratives, digestants, fats and oils, mineral and vegetable acids, alkalies, bitters, hematics, etc. Class II. Antiseptics, aromatics. Class III. Symptom medicines, including antispasmodics, antipyretics, anæsthetics, hypnotics, narcotics, motor excitants, motor depressants, cardiac stimulants, cardiac sedatives, diaphoretics, emetics, expectorants, cathartics, astringents, etc. A chapter is given to topical remedies—caustics, vesicants, rubefacients, emolients, demulcents, etc. A chapter of twenty-eight pages is devoted to prescription writing. The book is provided with a clinical and general index, thus adding much to the convenience of the student. S. E. H.

---

ELEMENTARY BANDAGING AND SURGICAL DRESSING, with directions concerning the immediate treatment of cases of emergency, for the use of dressers and nurses: by Walter Pye, F. R. C. S., late Surgeon to St. Mary's Hospital. Revised and in part re-written by G. Bellingham Smith, F. R. C. S., Surgical Registrar, Guy's Hospital. Seventh Edition. 218 pages, and numerous illustrations. Price, in cloth, 75 cents, net. W. B. Saunders, Publisher, 925 Walnut Street, Philadelphia. 1897.

Besides the many different forms of bandages, their method of application and uses, this little volume treats of the form and application of splints, of the simpler ways of dressing wounds, burns and scalds, the making of poultices and fomentations, of cupping, etc.; of the treatment in the first instance of accidents and emergencies, including fractures, hemorrhage, shock, collapse, fits, etc.; also of restoration from drowning and the treatment of cases of poisoning. It will serve the purpose for which it was intended most admirably. S. E. H.

